# An Inflammation Based Score Can Optimize the Selection of Patients with Advanced Cancer Considered for Early Phase Clinical Trials

**DOI:** 10.1371/journal.pone.0083279

**Published:** 2014-01-07

**Authors:** David J. Pinato, Chara Stavraka, Michael J. Flynn, Martin D. Forster, Séan M. O'Cathail, Michael J. Seckl, Rebecca S. Kristeleit, David Olmos, Samantha J. Turnbull, Sarah P. Blagden

**Affiliations:** 1 Wellcome Trust McMichael Clinical Research Facility, Imperial College London, Hammersmith Hospital, London, United Kingdom; 2 University College London Clinical Research Facility, University College London Hospital, London, United Kingdom; 3 Department of Oncology, Imperial College London. Hammersmith Campus, London, United Kingdom; 4 Royal Marsden Hospital and Institute of Cancer Research, Sutton, United Kingdom; University of Verona, Italy

## Abstract

**Background:**

Adequate organ function and good performance status (PS) are common eligibility criteria for phase I trials. As inflammation is pathogenic and prognostic in cancer we investigated the prognostic performance of inflammation-based indices including the neutrophil (NLR) and platelet to lymphocyte ratio (PLR).

**Methods:**

We studied inflammatory scores in 118 unselected referrals. NLR normalization was recalculated at disease reassessment. Each variable was assessed for progression-free (PFS) and overall survival (OS) on uni- and multivariate analyses and tested for 90 days survival (90DS) prediction using receiving operator curves (ROC).

**Results:**

We included 118 patients with median OS 4.4 months, 23% PS>1. LDH≥450 and NLR≥5 were multivariate predictors of OS (p<0.001). NLR normalization predicted for longer OS (p<0.001) and PFS (p<0.05). PS and NLR ranked as most accurate predictors of both 90DS with area under ROC values of 0.66 and 0.64, and OS with c-score of 0.69 and 0.60. The combination of NLR+PS increased prognostic accuracy to 0.72. The NLR was externally validated in a cohort of 126 subjects.

**Conclusions:**

We identified the NLR as a validated and objective index to improve patient selection for experimental therapies, with its normalization following treatment predicting for a survival benefit of 7 months. Prospective validation of the NLR is warranted.

## Introduction

The safety of individuals participating into phase I oncology studies is of paramount importance, where potentially high-risk investigational medicinal products (IMP) are administered for the first time in patients who may have limited life expectancy [1].

Stringent eligibility criteria are pre-defined to avoid the exposure of frail patients to potentially harmful or ineffective experimental treatments, as well as to protect trial results from possible inconsistencies in the assessment of the safety and tolerability of the IMP.

Despite this, the eligibility assessment of phase I candidates varies significantly as a function of the study protocol and relies mostly on subjective clinical parameters such as performance status (PS) and predicted life expectancy [2]. Although poor PS is a known predictor of mortality in cancer patients, concerns have been raised as to its true reliability in oncology trial patients [3] and there is disagreement as to whether subjects with “intermediate impairment” of their PS (ie. scoring PS = 2) can be safely offered the option of experimental treatments.

For these reasons, increasing research efforts have been made to qualify novel and more objective prognostic determinants in the phase I oncology patient population. A number of prognostic models have been proposed to improve the eligibility assessment and better predict their survival [4]. These models variously encompass predictors of worse outcome such as hypoalbuminemia, high tumour burden, elevated serum lactate dehydrogenase (LDH), lymphopenia as well as advanced PS [5], [6], [7], [8], [9].

However, there is substantial disagreement as to the optimal prognostic score as a result of the retrospective nature of some of the published studies and because of the lack of independent validation of the proposed algorithms [10]. Moreover, most of the studies inferring the utility of a given score in the screening process of phase I candidates have derived their prognostic information only from patients who actually received an IMP [11], [12] as opposed to unselected referrals prior to trial recruitment. As only about 30% of patients referred for phase I trials are ultimately offered treatment [13], this approach may have biased the screening of variables, limiting the generalizability of their prognostic power to the broader population of phase I referrals. As a result, none of the proposed prognostic scores are routinely incorporated in the design of phase I study protocols.

A second limitation of these scores is their inability to be dynamically used throughout the course of treatment to estimate treatment induced benefits and stratify individuals according to response. This is of greater consequence in early phase trials, as the qualification of reliable predictive markers of response may not only lead to a clearer identification of the 30–50% of patients achieving disease control following experimental treatments [7], [12], but also improve the detection of early pharmacodynamic effects, with major positive implications in the optimal dose selection of the tested compounds [14]. With many novel therapies causing disease stabilization without altering overall tumour size, there is a requirement for alternative methods for assessing IMP activity.

Inflammation is a critical component in the pathogenesis [15] as well as in the progression of cancer [16]. The presence of an acute phase reaction is a common event in cancer patients and results from the excess of pro-inflammatory cytokines such as interleukins (IL-1, IL-6, IL-8), tumour necrosis factor alpha (TNF-α) and interferons [17]. This systemic inflammatory response, that is deemed to reflect both disease activity as well as the host's innate response towards the tumour, has a causative role in determining most of the constitutional symptoms and signs reported by cancer patients including weight loss, anorexia, fatigue and cancer related anemia [18]. Systemic inflammation can be easily and reproducibly quantified in patients using a number of prognostic indices such as the neutrophil to lymphocyte (NLR) and platelet to lymphocyte ratio (PLR), both derived from inflammation-induced derangements in the full blood count.

The deterioration of these scores is a reliable predictor of survival in most solid tumors [19], independent of stage and histological subtype [20]. Moreover, treatment induced changes of the NLR have recently been qualified as predictors of response to treatment across different tumour types [21], [22], [23]. However, these scores have never previously been assessed in the phase I cancer population.

The aim of this study was therefore to comparatively test the NLR and PLR for their prognostic power in a series of unselected referrals to a phase I clinic. Additionally, we aimed to assess whether changes in these scores calculated at the pre-defined time of tumour reassessment can predict a significant survival advantage in patients treated in the context of phase I oncology trials.

## Methods

We conducted a retrospective analysis of all patients referred to the Hammersmith early phase trials unit with solid malignancies (Wellcome Trust McMichael Clinical Research Facility, WTMCRF) from January 2007 to December 2011. Patients were identified through clinic lists, paper and electronic medical records review. Complete demographic and treatment data including gender, age, tumour type and extent of metastatic spread, previous treatments, details of subsequent trial participation (eligibility, trial entry date) were collected together with the complete blood count, serum biochemical profile and PS. Clinical outcomes such as overall survival (OS, cancer specific) and the 90-days mortality rate (90DM) were calculated from the time of referral to our unit.

In patients who entered a phase I trial, progression-free survival (PFS) was calculated as time from the date of the first dose of IMP to the date of radiologically proven disease progression. Depending on protocol specific requirements, CT scan based tumour reassessment was carried out after 6–8 weeks from study baseline. Response to treatment was defined by a senior radiologist according to the Response Evaluation Criteria in Solid Tumours (RECIST 1.1) [24].

The NLR was calculated by dividing the absolute neutrophil count by the absolute lymphocyte count. NLR≥5 was considered elevated as previously described [25]. The same calculation was applied to derive the PLR, with 300 being the cutoff for positivity, in accordance with previously published literature [26]. Dynamic changes in the biomarker were defined as NLR normalization versus persistent abnormality as described before [22].

For a total of 8 patients participating in a phase I trial of an oral targeted agent, pre and post-treatment ^18^Fluorodeoxyglucose (FDG) positron emission tomography (PET) scans were available. The baseline scan was taken within 28 days from dosing, whereas follow up scans were taken following 2 cycles of treatment (8 weeks after the first dose of the IMP). All PET readings were performed by the same radiologist on PET-CT fused images, blinded to clinical outcomes. Changes in maximal standardize uptake values (SUVmax) were compared before and after treatment.

**Figure 1:**
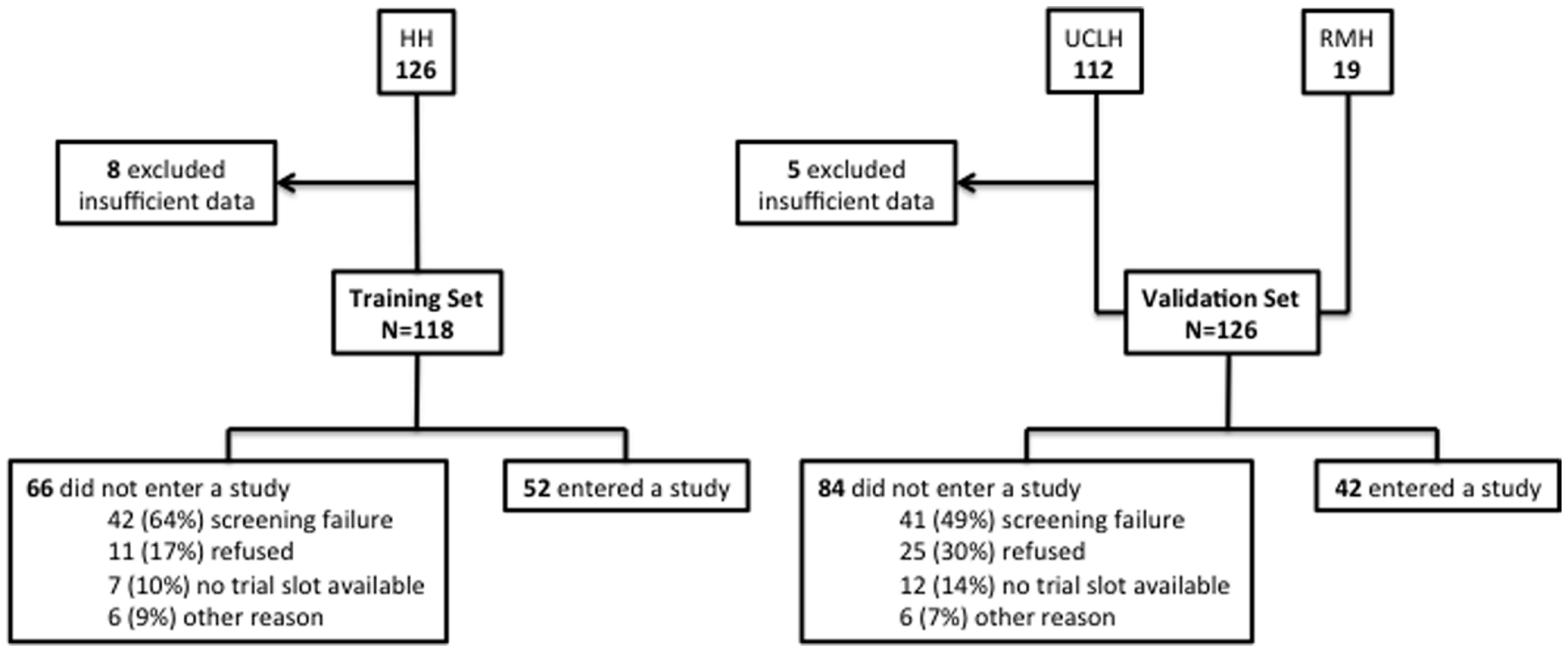
Study flow diagram illustrating patient disposition in the training and validation set.

We validated the significance of the tested prognostic variables in an independently collected set of data, using a separate cohort of 126 patients with similar characteristics. The validation cohort included a total of 107 patients presenting consecutively to the early phase clinical trials unit at the University College London Hospital (UCLH) from April 2010 to January 2012. A further set of 19 patients treated between October 2007 and February 2009 on a trial in collaboration with the Royal Marsden Hospital Drug Development Unit were included. A flow chart describing both patient cohorts is shown in **Figure 1**.

### Statistical Analysis

Pearson Chi-square test was used to assess for any associations between categorical variables. Univariate analysis of the different clinical factors associated with survival was carried out using Kaplan-Meier statistics and Log-rank test. Each factor was tested for its independent prognostic value using multivariate analysis according to Cox proportional hazard model using SPSS statistical package version 19 (IBM SPSS Inc., USA). A stepwise backward approach was used and variables with a p-value greater than 0.10 were removed from the model. The concordance index method (c index) was used to rank the different staging systems according to their capacity of discriminating patients according to outcome (OS). We assessed the effect of the candidate risk factors using the Cox model using R and the Statistical Analysis System (SAS, Cary, NC, USA) [27]. We used the rms packages of Dr Frank Harrell to identify a subset of predictors by backward elimination [28]. Where we assessed the predictive ability of a Cox proportional hazards model, we compared the actual survival outcomes of usable pairs of patients with the values of their estimated prognostic indices from the Cox model. Where the assessment of prediction of multiple biomarkers was performed, the c index was adjusted within the rms package for the over-optimism produced by modeling and assessment being done on the same data via comparison with 150 bootstrap samples. We quantified the improvement in the predictive ability of the top ranked prognostic score by calculating a new c index value reflecting the combination of prognostic variables. The c index of the resulting model was further internally validated by established bootstrapping techniques using 150 iterations. The receiver operating characteristic (ROC) curve method was used to compare the discriminative ability of candidate variables in predicting 90DM. All p-values presented are two-sided.

### Ethics Statement

All the patients included in this retrospective study had given explicit written consent for their information to be stored in the hospital database and used for research. All clinical investigations were conducted according to the principles expressed in the Declaration of Helsinki. Official approval for the use of retrospective data was granted by the Hammersmith Hospital Clinical Audit Office.

## Results

### Demographics

One hundred and twenty six patients were identified as new consecutive referrals to the WTMCRF at Imperial College London. Cases with insufficient follow up (n = 2) or with previous history of inflammatory disease or active concomitant infection at the time of referral (n = 6) were excluded. The clinicopathological variables describing our patient series are summarized in **Table 1**. Although baseline bloods were available for all patients, albumin was missing in 58 (49%) patients and LDH in 41 (34%) patients. In total, 96% of the patients were evaluable for the tested prognostic scores (NLR and PLR) whereas 98% were evaluable for ECOG PS.

**Table 1:** Patient characteristics.

		Training Set		Validation Set	
Baseline characteristic		n = 118 (%)	Median (range)	N = 126 (%)	Median (range)
**Gender**	Male	39 (33)	-	54 (43)	-
	Female	79 (67)		72 (57)	
**Age in years**	<65	65 (55)	63 (28–80)	82 (65)	62 (39–79)
	≥65	53 (45)		44 (35)	
**ECOG Performance Status**	0	33 (28)		18 (28)	
	1	53 (45)		83 (45)	
	2	20 (17)		21 (17)	
	3	9 (8)		4 (8)	
	Missing	3 (2)		-	
**Previous treatment lines**	0–2	63 (54)	2 (0–8)	75 (59)	2 (0–5)
	≥3	53 (46)		51 (40)	
**Tumour burden**	Locoregional disease only	10 (8)	2 (0–5)	6 (6)	2 (0–6)
	1–2 distant metastatic sites	87 (74)		64 (50)	
	≥3 distant metastatic sites	21 (18)		56 (44)	
**Areas of metastatic spread**	Liver	61 (52)		58 (46)	
	Lung	37 (31)		53 (42)	
	Bones	22 (19)		15(12)	
	Peritoneum	36 (30)		38 (30)	
	Extraregional lymphnodes	23 (20)		58 (46)	
	Brain	4 (3)		3 (2)	
	Other sites	21 (17)		19 (15)	
**Albumin**, g/L	<35 g/L	18 (70)	33 (13–43)	103 (70)	43 (32–49)
	≥35 g/L	42 (30)		12 (30)	
**Serum LDH**, IU/dL	<450 IU/dL	62 (52)	249 (46–4218)	36 (72)	264 (143–1816)
	≥450 IU/dL	15 (13)		14 (28)	
**Hemoglobin**, g/L	≥12 g/L	49 (41)	11.5 (8.2–14.4)	68 (54)	12.3 (8.0–17.0)
	<12 g/L	69 (59)		58 (46)	
**White blood cell count,** ×10^3^/liter	<10.5	95 (80)	7.3 (2–12)	97 (77)	6.8 (4.5–20.8)
	≥10.5	23 (20)		29 (23)	
**Platelet count,** ×10^3^/liter	<400	93 (79)	277 (69–626)	98 (77)	239 (98–474)
	≥400	25 (21)		28 (22)	
**Primary tumour group**	Gynaecological cancers	42 (35)		20 (16)	
	Gastrointestinal cancers	39 (33)		46 (37)	
	Breast cancer	18 (15)		10 (8)	
	Genitourinary cancers	5 (4)		9 (7)	
	Lung cancer/mesothelioma	4 (3)		10 (8)	
	Skin cancers/melanoma	4 (3)		4 (3)	
	Head and neck	3 (2)		23 (18)	
	Others	3 (2)		4 (3)	
**Overall Survival**, months			4.4 (0.2–39.0)		3.8 (0.5–43.4)

Training and Validation Cohorts.

In the training set, most patients were female (67%) and had evidence of distant metastases in at least one visceral site (91%). Twenty-five percent of the patients were PS 1–3. At the time of analysis 85 patients had died (72%). The cancer specific OS of the entire cohort was 4.4 months (range 0.2–39 months) and the overall ninety-day mortality rate was 41%. Fifty-two patients (44%) were treated within one of 7 phase I trials, the majority investigating molecularly targeted agents (71%). No patients were selected on the basis of target gene/protein expression. At the planned CT scan reassessment, one subject showed partial response (2%), 18 had disease stabilization (35%) whereas the remaining 33 had disease progression (63%). The median PFS in this subgroup was 1.7 months (0.2–18.7 months), while the median OS was 5.6 months (1.3–38.6 months).

### Relationship between Inflammatory scores and patient characteristics

According to the inflammatory scores, 36% of patients in the training set had an abnormal NLR whereas 33% had an abnormal PLR at baseline. At the time of disease reassessment, the NLR was recalculated and 34% of the treated subjects showed a worsening of their NLR index. An elevated NLR at baseline was associated with more advanced PS (p<0.001), presence of >2 sites of metastasis (p = 0.04), elevated LDH (p = 0.04), hypoalbuminemia (p = 0.002), anemia (p = 0.01) and younger age (p = 0.05). An elevated PLR was associated with anemia (p = 0.03) (**Table 2**).

**Table 2:** The relationship between clinicopathological factors and baseline inflammatory scores (NLR, PLR) in patients with advanced solid tumours considered for experimental treatments (Training Set).

Variable	NLR<5	NLR≥5	P	PLR<300	PLR≥300	P
**Gender**, M/F	24/46	14/29	**0.85**	28/46	10/29	**0.19**
**Age**, <65/≥65	34/36	29/14	**0.05** *	37/37	26/13	**0.09**
**ECOG PS**, 0–1/≥2	61/8	24/17	**<0.001** *	56/17	29/8	**0.84**
**N of metastatic sites**, <2/≥2	61/9	31/12	**0.04** *	63/11	29/10	**0.16**
**Liver metastases**, absent/present	38/32	17/26	**0.12**	36/38	19/20	**0.99**
**Lung metastases**, absent/present	50/20	26/17	**0.22**	50/24	26/13	**0.92**
**Bone metastases**, absent/present	61/9	32/11	**0.08**	60/14	33/6	**0.64**
**LDH**, <450/≥450 IU/L	42/6	20/9	**0.04** *	42/10	20/5	**0.93**
**Hb**, ≥12/<12 g/L	33/36	11/32	**0.01** *	34/39	10/29	**0.03** *
**Albumin**, ≥35/<35 g/L	35/35	9/34	**0.002** *	31/43	13/26	**0.37**

Marks an association reaching statistical significance (p<0.05).

### Inflammatory scores and survival

Univariate analysis of survival revealed albumin <35 g/L (p<0.001), LDH≥450 IU/L (p<0.001), advanced PS (p<0.001), haemoglobin <12 g/L (p = 0.01), number of previous systemic lines (p = 0.02), elevated NLR (p<0.001) as well as normalization of the NLR following treatment (p<0.001) as being significant predictors of OS, with hypoalbuminemia (p = 0.01), high LDH (p = 0.005), poor PS (p = 0.006), high risk NLR (p = 0.04) and NLR normalization (p = 0.03) qualifying as independent predictors following multivariate analysis.

**Figure 2:**
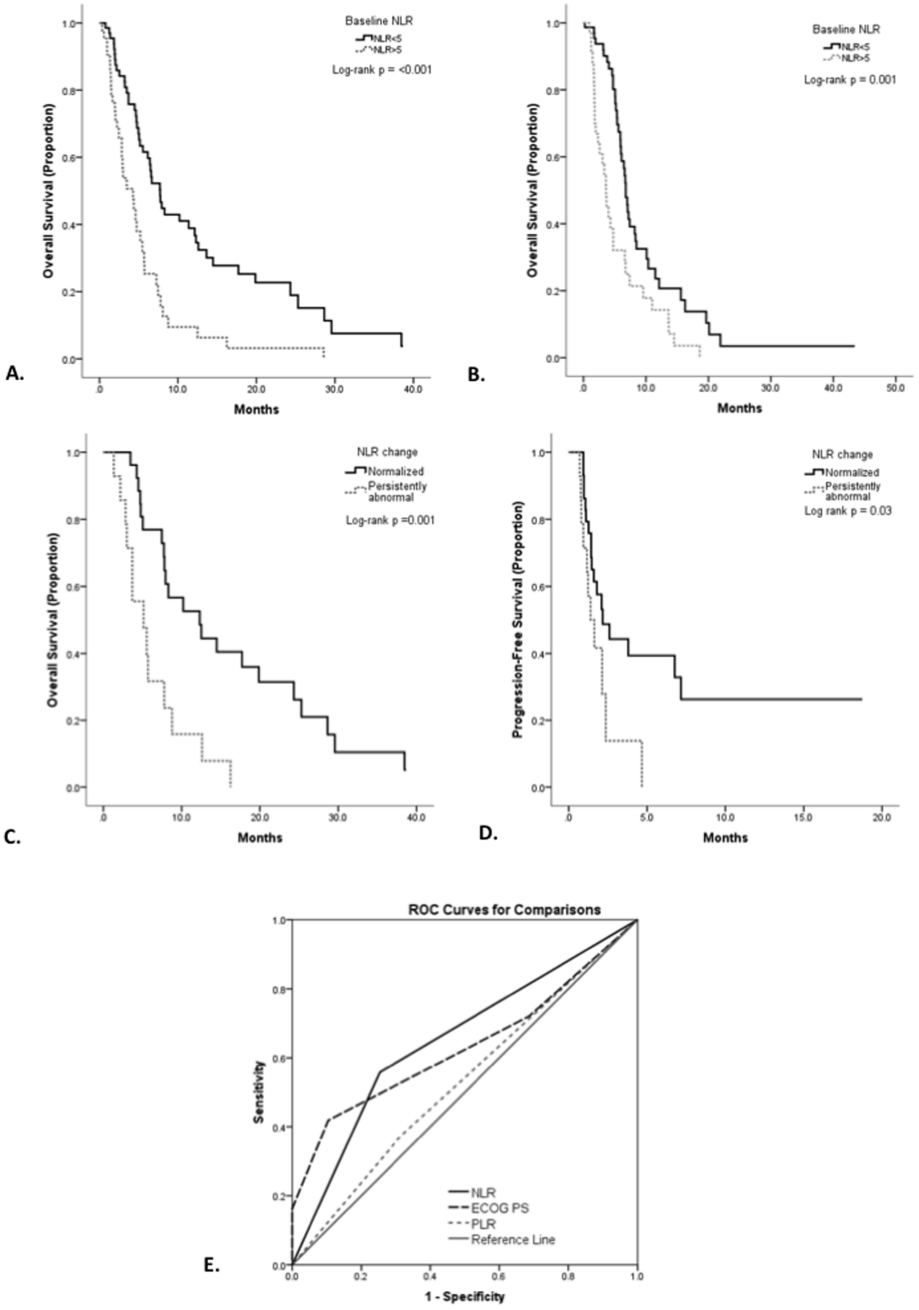
Kaplan Meier curve analysis showing that NLR≥5 predicts for poor OS in the training (Panel A) and in the validation set (Panel B). NLR normalization calculated at disease reassessment predicts for better OS (**Panel C**) and PFS (**Panel D**). Receiver operator curve for comparison of PS, baseline NLR and PLR for predicting 90 day survival (**Panel E**).

Patients in whom the NLR was ≥5 had a median OS of 4.2 months while patients with NLR<5 had a median OS value of 7.7 months. Normalization of the NLR at disease reassessment was associated with a 7 months improvement in OS (12.5 vs. 5.5 months) (**Figure 2**).

An association between LDH levels at presentation (p = 0.04) and NLR normalization following treatment (p = 0.04) and PFS was found and confirmed to have an independent predictive power at multivariate analysis (p = 0.009 and 0.008 respectively) (**Table 3**). Patients achieving NLR normalization at first reassessment had a median PFS of 3.8 months, while patients in whom the NLR remained persistently elevated or worsened following first reassessment had a median PFS of 1.3 months.

**Table 3:** Univariate and multivariate analysis of prognostic factors of overall and progression free survival (Training Set).

			OVERALL SURVIVAL					PROGRESSION-FREE SURVIVAL			
			UNIVARIATE ANALYSIS		MULTIVARIATE ANALYSIS			UNIVARIATE ANALYSIS		MULTIVARIATE ANALYSIS	
Variable		N = 118	Hazard Ratio (95% CI)	P-value	Hazard Ratio(95% CI)	P-value	N = 48	Hazard Ratio (95% CI)	P-value	Hazard Ratio (95% CI)	P-value
**Albumin**, g/L	≥35/<35 g/L	18/42	2.4 (1.5–3.9)	<0.001*	2.3 (1.2–4.4)	0.01*	23/26	1.8 (0.9–3.6)	0.08		
**LDH**, IU/L	<450/≥450	62/15	5.2 (2.5–10.7)	<0.001*	3.2 (1.4–7.3)	0.005*	35/7	3.6 (1.5–8.7)	0.04*	3.11 (1.3–9.0)	0.009*
**ECOG PS**	0–1/≥2	86/29	4.5 (2.6–7.6)	<0.001*	2.9 (1.4–6.4)	0.006*	45/3	2.3 (0.6–7.9)	0.12		
**N of metastatic sites**	<2/≥3	97/21	1.1 (0.6–1.9)	0.78			42/7	0.6 (0.2–1.8)	0.64		
**N of previous chemotherapy lines**	<2/≥3	65/53	1.7 (1.1–2.6)	0.02*			31/17	0.7 (0.3–1.6)	0.75		
**Hemoglobin**, g/L	≥12/<12	49/69	2.4 (1.5–4.0)	0.01*			22/27	1.7 (0.8–3.6)	0.13		
**NLR**	<5/≥5	70/43	2.5 (1.6–3.9)	<0.001*	2.0 (1.0–4.3)	0.04*	34/15	1.8 (0.9–3.6)	0.11		
**PLR**	<300/≥300	74/39	1.4 (0.9–2.2)	0.11			31/18	1.5 (0.8–3.1)	0.21		
**Delta NLR**	Normalized/persistently abnormal	32/16	3.6 (1.7–7.6)	0.001*	2.8 (1.1–7.4)	0.03*	32/16	3.0 (1.4–6.4)	0.04*	3.5 (1.3–7.2)	0.008*

Abbreviations: LDH, Lactate dehydrogenase; ECOG PS, Eastern Cooperative Oncology Group Performance Status; NLR, neutrophil to lymphocyte ratio; PLR, platelet to lymphocyte ratio: Delta NLR: NLR changes following 2 cycles of treatment as previously categorized by Kao et al. 2010 (Ref. 23). Associations reaching statistical significance (p<0.05) are marked with an asterisk (*). Categorization of LDH, haemoglobin and albumin was carried out using clinically employed cutoff values (Arkenau et al. 2008, Ref. 8). To avoid colinearity bias, the independent effect of NLR and Delta NLR was tested in two independent Cox models.

### Comparative performance of Prognostic Models

ROC curve analysis revealed ECOG PS (area under curve (AUC) = 0.63, 95% CI 0.53–0.77, p = 0.02), baseline NLR (AUC = 0.65, 95% CI 0.54–0.76, p = 0.007) but not baseline PLR (AUC = 0.53, 95% CI 0.42–0.64, p = 0.60) to significantly predict for 90DM (**Figure 2**).

The discriminatory capacity of each prognostic system was compared by means of Harrell's concordance index. The c-score value was calculated for each prognostic score. ECOG PS had a c-index score of 0.69 (95% CI 0.56–0.82), followed by the NLR 0.60 (95% CI 0.50–0.70) and PLR 0.53 (95% CI 0.42–0.64). Improvement of the discriminatory capacity of the first ranked prognostic variable was obtained by combining the NLR with ECOG PS, giving rise to a new c index of 0.72 (95% CI 0.59–0.83).

### Validation of Prognostic Models

The prognostic value and discriminative ability of inflammatory scores was further tested in an independent dataset composed of 126 patients with OS (median 3.8 months, range 0.5–43.4, p = 0.09), ECOG PS (20% PS>1, p = 0.14) and number of previous treatment lines (median 2, range 0–8, p = 0.34) similar to those described for the training set. The full clinicopathological profile of the validation cohort is described in **Table 1**.

In the validation set, advanced ECOG PS (HR 1.98 95% CI 1.2–3.1, p = 0.003), hypoalbuminemia (HR 4.3 95% CI 2.1–8.5, p<0.001) and elevated NLR at the time of referral (HR 2.2 95% CI 1.4–3.5, p = 0.001) were confirmed as univariate predictors of survival, whereas PLR>300 (p = 0.08) and number of metastatic sites (p = 0.69) did not retain prognostic value. Multivariate analysis of OS revealed that elevated NLR (HR 2.84 95% CI 1.6–5.0, p<0.001), hypoalbuminemia (HR 5.11, 95% CI 2.4–10.7 p<0.001) and poorer ECOG PS (HR 1.71, 95% CI 1.0–3.1 p = 0.08) independently predicted for worse OS. The discriminatory capacity of the NLR, as assessed by the c index, was 0.63 (95% CI 0.51–0.76) whereas the calculated c score for ECOG PS was 0.65 (95% CI 0.54–0.76). An improvement in the discriminative ability of PS was confirmed in the validation set when combined with the NLR, deriving a resulting c score of 0.70 (95% CI 0.59–0.81). As shown in **Table 4**, an elevated NLR predicted for significantly worse survival outcomes in patients with preserved as well as advanced ECOG PS in both training and validation set.

**Table 4:** Integration of the NLR with ECOG PS in the prediction of OS (Training and Validation Set).

Training Set#					
ECOG PS	NLR	N	Median OS (months)	95% CI (months)	P-value§
0	<5	24	13.6	0.5–32.5	0.01*
	≥5	9	7.5	1.5–13.3	
1	<5	37	6.5	4.5–8.5	
	≥5	15	5.4	3.5–7.4	
2–3	<5	8	2.5	0.5–6.2	
	≥5	17	2.2	1.0–3.5	

Validation Set##					
ECOG PS	NLR	N	Median OS (months)	95% CI (months)	P-value§
0–1	<5	36	6.7	6.1–7.4	0.05*
	≥5	24	4.7	3.5–5.9	
2–3	<5	4	4.7	4.4–5.0	
	≥5	9	1.8	1.7–1.8	

Chi-square test of equality of survival distributions for the different NLR categories.

^#^ Patients with PS 2 and 3 were considered together due to the small number of patients with PS = 3 (n = 9),

^##^ Patients were dichotomized as “favourable PS” (ie. 0–1) versus “poor PS” (ie. 2–3) due to limited sample size.

Marks an association reaching statistical significance (p<0.05).

### Inflammatory scores and FDG-PET response

**Figure 3:**
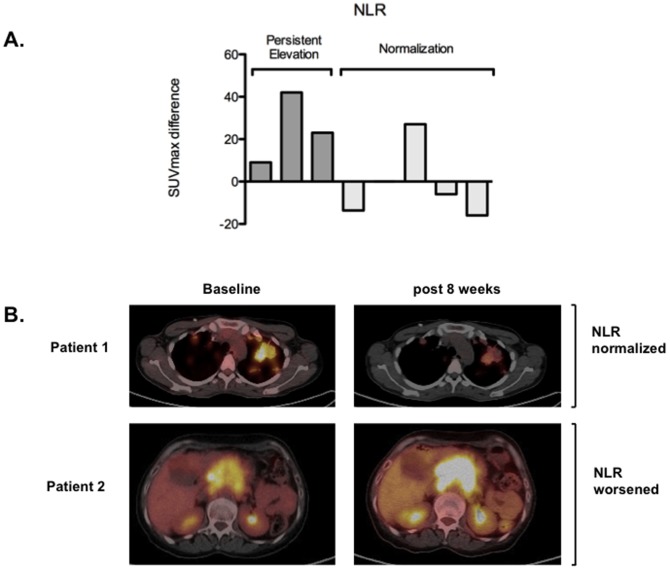
Exploratory subanalysis investigating the relationship between NLR normalization and ^18^FDG-PET SUVmax in patients treated with a molecularly targeted IMP (n = 8). **Panel A:** Waterfall plot showing individual metabolic responses in patients with normalized versus persistently elevated NLR following treatment. **Panel B:** Representative PET-CT fused axial images obtained at screening and after 8 weeks of treatment with an oral targeted agent. In patient 1 a 15% reduction of SUVmax in the region of interest is associated with NLR normalization following treatment. In patient 2 metabolic progression of disease, with a 40% increase in SUVmax is associated with worsening of the NLR at the time of disease reassessment.

An exploratory analysis investigating the association between changes in the NLR following treatment and metabolic response measured by ^18^FDG PET-CT was undertaken in 8 subjects in whom pre and post treatment ^18^FDG PET-CT scans were used as a pharmacodynamic endpoint. Changes in SUVmax compared before and after treatment and are summarized in a waterfall plot, where each column is representative of the SUVmax change in each individual patient (**Figure 3A**).

All patients displaying metabolic progression of disease (3/3) were categorized as having a persistently elevated NLR following treatment. Conversely, the achievement of NLR normalization was associated with an overall decrease or stability in SUVmax values in 3/4 subjects (**Figure 3B**).

## Discussion

Patients with advanced solid malignancies who have been referred for consideration of phase I trial entry represent a uniquely heterogenous population of individuals with different types of cancer, exposure to multiple lines of previous therapy and a life expectancy that rarely exceeds 9 months [1].

Exhaustion of standard treatments, adequate performance status and organ function are generally the only criteria that guide accrual into early phase trials. However these parameters are insufficient predictors of overall survival, or as early means of identifying patients who are deriving benefit from experimental treatments [2].

In this study we aimed to qualify the clinical value of simple inflammatory related scores such as the NLR and PLR both as predictors of the overall prognosis of our patients and as dynamic markers that could be used to stratify trial participants according to their response to treatment.

We have shown that the NLR is an independent predictor of OS, PFS and early mortality in an unselected series of patients with advanced cancer referred to a phase I service. Interestingly, our data show that patients in whom the NLR normalized at the moment of planned disease reassessment had a survival gain of 7 months compared to individuals remaining in or worsening to the “high risk” category throughout treatment. This follows the observation made across several other studies in which NLR changes induced by treatment were an independent early predictor of treatment benefit [21], [23], [29], [30].

There is compelling evidence in the literature showing that the presence of a systemic inflammatory reaction is predictive of worse outcome in patients with cancer, independent from tumour site and stage [20]. Among the several methods used to measure systemic inflammation, the NLR and PLR are the most used parameters [31] in conjunction with the Glasgow Prognostic Score (GPS) that takes into account hypoalbuminemia and elevation of C-reactive protein (CRP) [32].

A raised NLR reflects a combined state of neutrophilia and relative lymphopenia consequential to the systemic release of proinflammatory cytokines by cancer cells or by the host's innate immune system as part of a coordinated anti-tumour response. Lymphopenia is a known predictor of mortality in cancer patients [33] and part of its detrimental effect on prognosis has to be found in the impairment of the CD8+ cytotoxic immune system branch, with a consequential reduction of the immune-mediated antitumour response [34]. An increased NLR also reflects sustained angiogenesis and proliferative potential of tumour cells, two unfavorable hallmarks of cancer [26].

In our study cohort, patients with a raised NLR had a significantly poorer PS, higher LDH, more advanced disease and a higher prevalence of anemia, confirming that elevation of the NLR indicates a more aggressive clinical phenotype. This is not an unexpected finding since the presence of a systemic inflammatory response is known to underlie most of the clinical manifestations of advanced cancer including fatigue, cachexia and nutritional decline [17]. Moreover, in an exploratory subanalysis of 8 subjects in whom pre and post treatment FDG-PET scans were used as a pharmacodynamic endpoint, we found that all patients displaying metabolic progression of disease were categorized as having a persistently elevated NLR following treatment. Conversely, the achievement of NLR normalization was associated with an overall decrease or stability in SUVmax values in 3 out of 4 subjects (**Figure 2**). Such observation, although preliminary in nature, seems to further substantiate the link between disease activity and worsening of the inflammatory scores.

In our screening of prognostic traits the baseline NLR ranked as the most informative variable in predicting early mortality and followed patient's PS as the second most accurate predictor in estimating OS. Moreover, addition of the NLR to PS significantly increased the discriminative ability of PS alone. In particular, we noted that the an elevated NLR independently predicted for worse survival outcome in patients with preserved as well as more advanced PS (**Table 4**). This finding holds significant implications in the screening process of phase I candidates, as adequate PS (ECOG 0–2) and a predicted life expectancy exceeding 90 days are the most clinically utilized criteria in assessing the eligibility of patients with advanced cancer considered for early phase trials. Based on our findings, the NLR could therefore be usefully integrated with PS to increase the overall accuracy of prognostic prediction in such a heterogeneous patient population.

The prognostic impact of biomarkers of systemic inflammation has been left relatively unaddressed in early phase clinical trial patients, despite previous reports highlighting the prognostic value of individual determinants of ongoing inflammatory reaction such as leukocytosis, neutrophilia, lymphopenia, thrombocytosis and hypoalbuminemia in this patient population [5], [35], [36], [37].

One of the major limitations in the assessment of novel prognostic models in patients with advanced cancer is the single-institutional and retrospective nature of most studies [10], where survival and eligibility rates can be inherently different across institutions depending on the efficiency of the referral process within each institution, the availability of clinical trial slots and the presence of trial-specific eligibility criteria. A methodological strength of our study comes from the evaluation of the NLR and PLR by means of cross-validation in an independently collected set of patients in an attempt to reduce sampling bias and overcome potential systematic error relating to the presence of missing data [38]. Interestingly, our analysis showed that the NLR and its dynamic changes following treatment are independent predictors of survival in both the training and the validation set, therefore strengthening the generalizability of this observation. Based on our results, no prognostic role could be inferred for the PLR in patients considered for phase I trials, suggesting that the NLR is a more accurate biomarker of systemic inflammation.

In our study we could not assess the prognostic value of the Glasgow Prognostic Score, since CRP was not routinely measured in the majority of our patients. Because of the retrospective nature of our study, we were unable to validate the NLR prospectively, an approach that may be suggested in future studies, especially in light of the relatively small sample size of our patient cohorts compared with previously published retrospective studies involving more than 2000 phase I study participants [39]. The significant amount of missing data emerging for some of the variables we analyzed needs to be taken into consideration as a limitation to our study, strengthening the need for further prospective validation of the NLR before systemic inflammation can be confidently applied in the clinical arena. A further advantage of inflammation based scores that will have to be explored in prospective studies relies on their potential role in predicting toxicity from anticancer treatments, which is largely contingent on an inflammation induced impairment of cytochrome 3A activity [40] as well as patients' nutritional decline [41].

In conclusion, we have shown that inflammatory related changes in common laboratory markers such as the NLR are easy to compute, universally available, inexpensive and reproducible biomarkers that can be used in the prognostic assessment of potential phase I candidates as well as in the prediction of clinical benefit from experimental treatments.

ECOG-PS is a largely utilized screening tool and remains the gold standard prognostic determinant in patients with advanced cancer [6], [36], [37], [42]. Here we provide preliminary evidence that the NLR can be easily combined with ECOG-PS to achieve an improved and more objective estimation of patient's prognosis. Given that our study included consecutive phase I referrals, our *ad hoc* sub-analysis was not powered to explore the relationship between each ECOG-PS stratum and the NLR, leading to the need to combine prognostic strata and to subgroup patients in broader categories including “favorable” (ie. 0–1) versus “poor” (ie. 2–3) PS, a limitation that should addressed prospectively.

Indeed, the magnitude of the prognostic improvement emerging by the combination of ECOG-PS and NLR is modest based on our c-index analysis. Nonetheless, our study promotes the concept of a concurrent assessment of patients' PS and systemic inflammatory status, two independent and non-mutually exclusive prognostic domains whose combined evaluation should be further explored in future, adequately powered clinical studies. Taken together, our results promote the use of the NLR as a universally available biomarker to optimize the eligibility assessment of patients with advanced cancer considered for phase I trials and serve as an early predictor of response to experimental treatments.
